# Single-Cell RNA Sequencing Reveals LEF1 as a Prognostic Biomarker for Poor Outcomes in Oxaliplatin-Resistant Colorectal Cancer

**DOI:** 10.1155/humu/6705599

**Published:** 2025-08-06

**Authors:** Pin Huang, Ke Guo, Jiancheng Tu, Jian Fang, Liang Zhou, Xiagang Luo, Hubin Xu

**Affiliations:** ^1^Department of General Surgery, The Affiliated Zhangjiagang Hospital of Soochow University, Suzhou, Jiangsu, China; ^2^Department of General Surgery, The Second Affiliated Hospital of Nanjing Medical University, Nanjing, Jiangsu, China

**Keywords:** colorectal cancer, lymphoid enhancer-binding factor 1 (LEF1), oxaliplatin resistance, prognostic biomarkers

## Abstract

Colorectal cancer (CRC) is a leading cause of cancer-related morbidity and mortality worldwide. Despite the efficacy of oxaliplatin-based chemotherapy (CT) in CRC treatment, CT resistance remains a major obstacle to successful patient outcomes. Epithelial–mesenchymal transition (EMT), a key cellular process in cancer metastasis, plays a pivotal role in resistance to CT. The tumor microenvironment (TME), particularly cancer-associated fibroblasts (CAFs), is known to contribute to EMT and therapy resistance. Here, we employ single-cell RNA sequencing (scRNA-seq) to analyze primary CRC tumor samples from patients undergoing CT and nonchemotherapy (nCT) treatments. Our study identifies specific epithelial cell clusters resistant to oxaliplatin, elucidating the molecular pathways involved in EMT and resistance. Furthermore, we explore the role of CAF subpopulations in promoting resistance within the TME. Our findings highlight the importance of functional immune profiling and genomic analyses in identifying potential biomarkers for predicting CT responses and improving personalized treatment strategies. This work provides new insights into the molecular mechanisms of oxaliplatin resistance in CRC and supports the development of novel immune-based therapeutic approaches to enhance patient outcomes.

## 1. Introduction

Colorectal cancer (CRC) is one of the most common and deadly cancers worldwide, ranking third in incidence and second in mortality [1]. Oxaliplatin, a DNA synthesis inhibitor, is the most widely used first-line chemotherapy (CT) for CRC. It induces DNA cross-links, blocking DNA replication and transcription, ultimately leading to cell death [2]. However, CT resistance is a major cause of treatment failure and disease progression in many CRC patients. Therefore, understanding the molecular mechanisms of CT resistance and exploring new therapeutic strategies to improve patient survival have become urgent clinical priorities.

Epithelial–mesenchymal transition (EMT) plays a crucial role in the initiation, progression, metastasis, and resistance to cancer therapies [3]. Tumor-associated stromal cells can activate EMT in cancer cells through various pathways, enhancing resistance [4]. Among these, cancer-associated fibroblasts (CAFs) are key players [5]. In the tumor microenvironment (TME), CAFs induce EMT in adjacent cancer cells through paracrine or juxtacrine signaling mechanisms [6]. However, due to the heterogeneity of CAFs in terms of their origin and function, the mechanisms by which specific CAF subtypes induce EMT in CRC cells and contribute to CT resistance remain unclear and require further study [7].

Single-cell RNA sequencing (scRNA-seq) offers a more in-depth understanding of CT responses in solid tumors, revealing the heterogeneity of resistant cells and their TME [8, 9]. This technique helps uncover resistance mechanisms and identify potential targets for personalized therapy. In this study, we analyzed scRNA-seq data from primary CRC tumors of CT and nonchemotherapy (nCT) patients. We identified oxaliplatin-resistant epithelial cell clusters and elucidated the molecular mechanisms of EMT in these epithelial subpopulations and two CAF subpopulations involved in resistance. We also identified the transcription factor LEF1 as a key driver of resistance. Our findings suggest that LEF1 induces EMT through the TGF-*β* signaling pathway, contributing to CRC CT resistance to oxaliplatin.

## 2. Materials and Methods

### 2.1. Collection of scRNA-seq Data

We retrieved scRNA-seq datasets of primary CRC from the Gene Expression Omnibus (GEO) database, with the accession numbers GSE225857 [10] and GSE245552 [11], corresponding to CT-treated and untreated (nCT) CRC samples, respectively. From the GSE225857 dataset, we selected five primary tumor samples from CRC patients who had received FOLFOX or FOLFOXIRI CT. Additionally, from the GSE245552 dataset, we selected 16 primary tumor samples from untreated CRC patients.

### 2.2. Data Quality Control and Cell Annotation

Single-cell data were processed using the CreateSeuratObject function from the Seurat package (V4.4.0), with parameters set as min.cells = 5 and min.features = 300, to generate Seurat objects [12]. The quality control criteria were as follows: (1) The number of genes detected per cell ranged from 300 to 8000; (2) the percentage of mitochondrial genes did not exceed 25% of the total UMIs; (3) the percentage of ribosomal genes did not exceed 30% of the total UMIs; and (4) cells with more than 1000 UMIs were retained, and the top 3% of cells with the highest UMIs were excluded.

To correct for batch effects across samples, we integrated the single-cell data using the Harmony package (v1.2.0) [13]. The data were then clustered into 21 distinct clusters using the FindClusters function (with a resolution of 0.4 and based on the first 25 principal components). Uniform manifold approximation and projection (UMAP) was employed for data visualization. Marker genes for each cluster were identified using the FindAllMarkers function from Seurat (with parameters set as min.pct = 0.1 and logfc.threshold = 1). Finally, all cells were annotated as 10 major cell types using the CellMarker database.

### 2.3. InferCNV

We performed chromosome copy number variation (CNV) analysis on all epithelial cells using the InferCNV (V1.3.3) package (https://github.com/broadinstitute/inferCNV), with myeloid cells as the reference. The initial CNV score for each cell was calculated, and the results were visualized as a heatmap, where red and blue indicate amplifications and deletions of chromosome regions, respectively. Finally, nonmalignant epithelial cells were filtered out using the average CNV score of myeloid cells as the threshold.

### 2.4. Identification of Oxaliplatin-Resistant Epithelial Cells

The R package Scissor (V2.0.0) represents an innovative approach. It utilizes the phenotypic information (including disease stage, tumor metastasis, treatment response, and survival outcomes) collected from bulk transcriptomic profiles to identify the cell subpopulations most strongly associated with phenotypes within single-cell data. The GDSC2 database offers the processed bulk transcriptomic profiles of 805 tumor cell lines, along with the corresponding oxaliplatin IC50 values [14]. Cell lines were classified as oxaliplatin-resistant or oxaliplatin-sensitive according to the upper and lower one-third cut-off values of the oxaliplatin IC50 values.

In our study, we incorporated three data sources as inputs for the Scissor algorithm: (1) A single-cell expression matrix of 13,018 malignant epithelial cells derived from CRC. (2) A bulk expression matrix of tumor cell lines sourced from the GDSC database. (3) The oxaliplatin IC50 phenotypic information for each tumor cell within the GDSC.

By leveraging these inputs, the Scissor algorithm was applied to selectively identify oxaliplatin-resistant epithelial cells. Subsequently, the output of the Scissor algorithm was projected onto a UMAP plot for further visualization, thereby uncovering a notable aggregation of oxaliplatin-resistant cells within specific cell clusters.

### 2.5. Monocle2 Pseudotime

We used Monocle (V2.30.1) to infer the pseudotemporal dynamics of malignant epithelial cells in the CRC scRNA-seq dataset [15]. First, a CellDataSet object was created using the newCellDataSet function, and variable genes were identified using the VariableFeatures function. Next, dimensionality reduction and cell ordering were performed using the DDRTree method, followed by sorting the cells using the orderCells function.

### 2.6. Intercellular Communication Analysis

To explore the communication patterns between oxaliplatin-resistant epithelial cells, we utilized the CellChat R package (V1.6.1) to infer potential intercellular communication networks based on ligand–receptor interactions [16]. First, we imported the CT and nCT data, using the normalized Seurat data as the CellChat object. Next, the filterCommunication function (min.cells = 10) filtered out subclusters with low communication counts. Finally, the computeCommunProbPathway function in CellChat was applied to calculate the communication probabilities of signaling pathways, and the aggregateNet function was used to compute the aggregated intercellular communication network, enabling a comparison of the communication networks between the CT and nCT groups.

To investigate the interactions between ligands, receptors, and target genes in malignant epithelial cells and CAFs, we used NicheNet (V2.1.0) [17]. For ligand–receptor interaction analysis, we considered genes expressed in more than 10% of clustered cells. Specifically, we extracted the Top 30 differentially expressed ligands and their corresponding target genes, along with the Top 20 receptors from both “sender” and “receiver” cells, for relative activity analysis. The Top 30 ligands were assessed using the area under the precision–recall curve (AUPR), and an AUPR heatmap was generated. Additionally, heatmaps for ligand–target interactions and ligand–receptor interactions were constructed to visualize the differentially expressed ligands, target genes, and receptors.

### 2.7. SCENIC Analysis

SCENIC is a tool that reconstructs gene regulatory networks and identifies stable cellular states from scRNA-seq data. To evaluate the enrichment of key transcription factors and the activity of regulons in each malignant epithelial cell subcluster, we performed the analysis using the pySCENIC package (V0.12.1) [18]. The human genome hg38 was used as the reference genome, and motif annotations from v9 nr.hgnc were employed for genomic locus analysis. The regulon specificity score (RSS) for all transcription factors annotated by HGNC was calculated to identify regulatory factors specific to each cluster. Finally, the visualization results display only the Top 15 transcription factors based on their RSS rankings.

### 2.8. GO, KEGG, and Hallmark Pathway Enrichment Analyses

We used FindMarkers (with parameters set to logfc.threshold = 0.25 and min.pct = 0.1) to identify differentially expressed genes, followed by pathway enrichment analysis using the gseGO and gseKEGG functions from the clusterProfiler package (V4.1.0) [19]. To present the enrichment results clearly, we visualized them using GseaVis (V0.1.0). Additionally, we calculated the pathway activity scores for hallmark gene sets from the MSigDB database using GSVA (V1.5.0) for each cell. The resulting scores were visualized in heatmaps using pheatmap (V1.0.12).

### 2.9. Cell Lines and Cell Culture

The HCT116 human colon cancer cell line resistant to oxaliplatin was purchased from Shanghai Zhongqiao Xinzhao Biotechnology Co., Ltd. and authenticated by short tandem repeat (STR) analysis. The cells were cultured following the guidelines provided by the supplier. Specifically, HCT116/L cells were maintained in DMEM (Gibco) supplemented with 10% fetal bovine serum (FBS, Vazyme, Nanjing, China), 1% penicillin–streptomycin solution (Beyotime, Shanghai, China), and 8 *μ*g/mL oxaliplatin. All cells were kept in a humidified incubator at 37°C with 5% CO₂ and 95% air.

### 2.10. Plasmids and Transfection

Short hairpin RNA (shRNA) targeting LEF1 and the negative control shRNA (sh-NC) were obtained from Proteinbio Corporation (Nanjing, China). The plasmid expressing Flag-tagged LEF1 was purchased from Meiling Biotech (Wuhan, China). When cells reached 50%–70% confluency, RNA or plasmids were transfected into the cells using Lipofectamine 3000 (Invitrogen, Carlsbad, United States) according to the manufacturer's instructions. The sequences of the siRNAs are as follows: LEF1-shRNA, 5⁣′-GTTGCTGAGTGTACTCTAA-3⁣′;Sh-NC, 5⁣′-TTCTCCGAACGTGTCACGT-3⁣′ [20].

### 2.11. CCK-8 Assay

Then, 48 h after cell transfection, cell suspensions were prepared using either serum-free medium or medium containing 1% serum. Approximately 2000 cells were seeded into each well of a 96-well plate, and after about 24 h, the cells were cultured in medium containing 10% serum. At 0, 24, 48, 72, and 96 h, 10 *μ*L of Cell Counting Kit-8 (CCK-8, #A311-01, Vazyme) reagent was added to each well. The optical density of the cells was measured at a wavelength of 450 nm using a microplate reader (BioTek, United States), and the cell growth curve was plotted.

### 2.12. EdU Incorporation Assay

The EdU kit (#C0075, Beyotime) was purchased from Beyotime Biotechnology. A suspension of approximately 20,000 cells per well in serum-free medium was seeded into a 48-well plate. After 24 h of incubation, the medium was replaced with complete medium containing 10% FBS. Once the cells reached 80% confluence, EdU was added to each well and incubated with the cells for 2 h. Following incubation, cells were fixed with 4% paraformaldehyde at room temperature for 30 min. The cells were then permeabilized with 0.3% Triton X-100 for 15 min. The EdU reaction mixture was prepared according to the kit instructions and incubated with the cells in the dark for 30 min. Finally, the cells were stained with 4⁣′,6-diamidino-2-phenylindole (DAPI) for 10 min, and imaging was performed under a fluorescence microscope in a dark room.

### 2.13. Transwell Assays

Cell migration and invasion assays were conducted using 6.5-mm Transwell devices (polycarbonate membrane inserts with 8.0-*μ*m pores, #CLS3422, Corning). For the migration assay, 750 *μ*L of medium containing 10% FBS was added to the lower chamber, and 200 *μ*L of serum-free cell suspension (8 × 10^4^ cells/mL) was seeded into the upper chamber. The cells were incubated at 37°C for at least 48 h, fixed with 4% paraformaldehyde, and stained with crystal violet solution. The stained cells on the underside of the membrane were then counted by gently wiping off the cells from the upper chamber and visualizing under an optical microscope. For the invasion assay, the upper chamber was pre-coated with 50 *μ*L of a diluted 1:10 Matrigel matrix (#356234, Corning) before seeding the cells, with the remaining steps identical to the migration assay.

### 2.14. Western Blot

HCT116 oxaliplatin-resistant cells in the logarithmic growth phase were washed with PBS, followed by protein extraction using RIPA lysis buffer containing 1% PMSF and phosphatase inhibitors. The protein concentration in each group was determined using a BCA kit (Beyotime, China). Equal amounts of approximately 30 *μ*g protein were loaded onto a 10% SDS-PAGE gel. After transferring to a PVDF membrane, the membrane was blocked for 2 h at room temperature using a 5% BSA solution. The membrane was then incubated overnight at 4°C with primary antibodies: anti-E-cadherin (1:1000, abcam, ab40772), anti-N-cadherin (1:1000, abcam, ab76011), anti-Vimentin (1:1000, abcam, ab20346), anti-*β*-actin (1:5000, Sigma Aldrich #A5441), and anti-LEF1 (1:5000, proteintech, 28540-1-AP). After 1 h of incubation with the corresponding secondary antibodies, chemiluminescent detection was performed using a gel imaging system.

### 2.15. Statistical Analysis

All statistical analyses and data processing were conducted using R (V4.3.2), Python (V3.7), and GraphPad Prism 9.0. Normally distributed data are expressed as mean ± standard deviation (mean ± SD), while skewed data are expressed as median (interquartile range, median [IQR]). Statistical tests used include the independent *t*-test and Wilcoxon rank-sum test, with significance set at *p* < 0.05 (ns, *p* ≥ 0.05; ⁣^∗^*p* < 0.05; ⁣^∗∗^*p* < 0.01; and ⁣^∗∗∗^*p* < 0.001).

## 3. Results

### 3.1. Identification of scRNA-seq Clusters in Primary CRC Under Different CT Conditions and Oxaliplatin-Resistant Clusters

**Figure 1:**
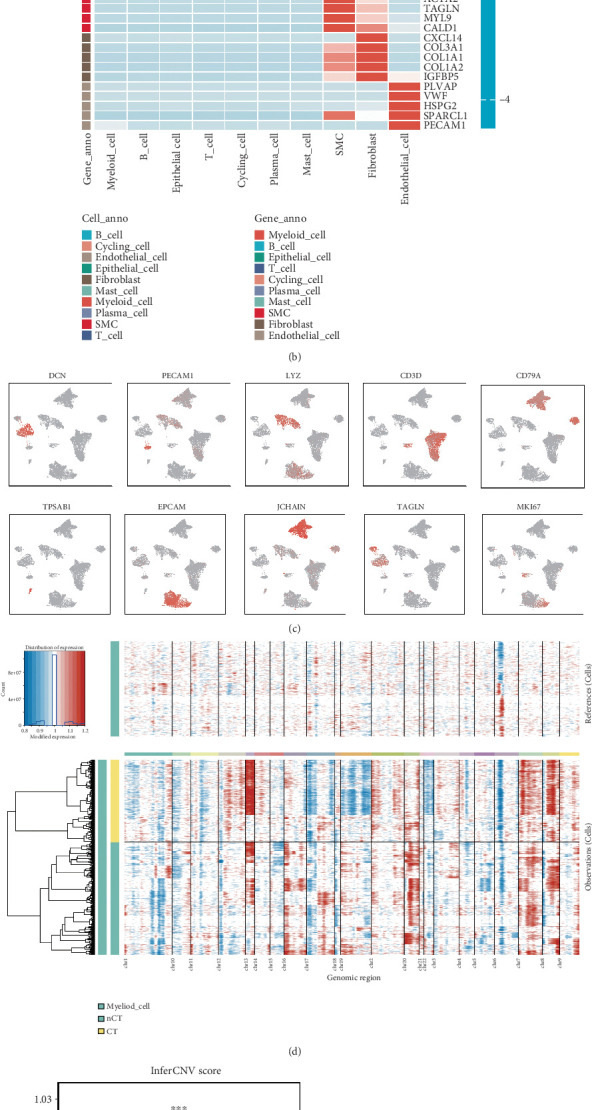
Single-cell transcriptomics atlas of CRC with and without chemotherapy. (a) The UMAP plots of scRNA-seq were colored according to sample origin, patient, cluster, and cell type. (b) The heat map illustrates the expression levels of marker genes across major cell types. (c) The UMAP plot shows the distribution of marker genes. (d) Chromosomal heat maps of CNVs in epithelial cells, with red for amplification and blue for deletion. (e) Boxplot for CNV scores (CT, nCT, and myeloid cell). ⁣^∗^*p* < 0.05, ⁣^∗∗^*p* < 0.01, and ⁣^∗∗∗^*p* < 0.001, Student's *t*-test. (f) UMAP plots of malignant epithelial cells, colored by sample origin (CT and nCT). (g) UMAP plots of malignant epithelial cells, with 16 clusters. (h) The UMAP plot shows the oxaliplatin-resistant epithelial cells in the CT and nCT groups predicted by the GDSC database.

First, we collected single-cell data from 21 CRC patients, including 5 CT-treated and 16 untreated (nCT) cases. After a series of quality controls, we generated a high-quality gene expression matrix for a total of 102,608 CRC cells, with 41,705 cells from the CT group and 60,903 cells from the nCT group. We then used Harmony for batch correction based on patient IDs and cell integration, identifying 21 cell clusters (Figure 1a). Based on classic cell markers (Figure 1b,c), we identified 10 major cell types (Figure 1a), including epithelial cells (20,067 cells), B cells (5199 cells), proliferating cells (1736 cells), endothelial cells (2120 cells), fibroblasts (7324 cells), mast cells (1792 cells), plasma cells (23,061 cells), smooth muscle cells (2223 cells), and T cells (28,837 cells).

To identify high-quality malignant epithelial cells, we used InferCNV to calculate the CNV scores for each epithelial cell with myeloid cells as the reference. The heatmap of chromosomal mutations annotated by CT status revealed different mutation patterns between CT and nCT (Figure 1d), with higher CNV scores in the nCT group compared to the CT group (Figure 1e), indicating epithelial cell heterogeneity between the groups. Using the average CNV score of myeloid cells as a threshold, we classified the epithelial cells into malignant (13,018 cells) and nonmalignant (7043 cells) types. Among these, the malignant epithelial cells in the CT and nCT groups were 4987 and 8031, respectively (Figure 1f). Through further clustering, we identified eleven subclusters of malignant epithelial cells (Figure 1g).

To explore oxaliplatin-resistant tumor epithelial cells in CRC, we used the Scissor algorithm to integrate the bulk sequencing phenotype data from tumor cell lines in the GDSC database with our integrated CRC single-cell data, aiming to decode the potential biological functions of each single-cell cluster at the phenotype level. The Scissor algorithm revealed significant clustering of oxaliplatin-resistant cells in Malignant_EP_04, 06, 07, and 10. However, only Malignant_EP_04 showed the resistant phenotype in the CT group (Figure 1h), suggesting that Malignant_EP_04 possesses the highest potential for oxaliplatin resistance.

### 3.2. Characterization of Oxaliplatin-Resistant Clusters

**Figure 2:**
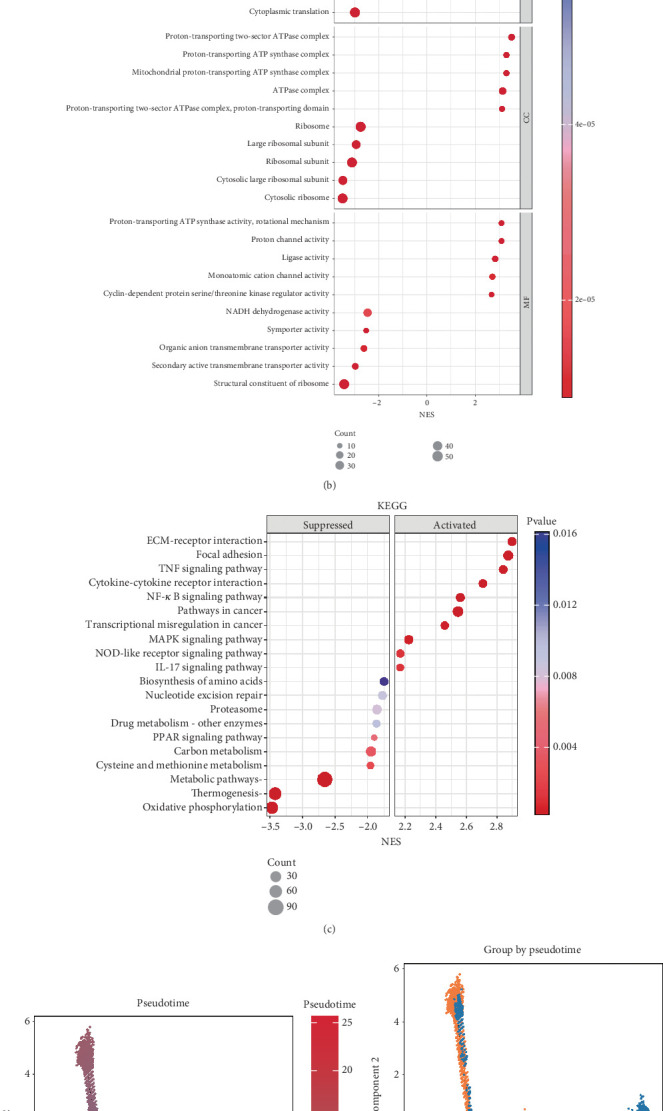
The characterization of epithelial cells resistant to oxaliplatin. (a) The heatmap shows hallmark pathway enrichment in a subcluster of malignant epithelial cells. (b) The dot plot shows the GO enrichment of the oxaliplatin-resistant subcluster in the CT group. (c) The dot plot shows the KEGG enrichment of the oxaliplatin-resistant subcluster in the CT group. (d) The UMAP plot shows the potential trajectories of all malignant epithelial cells colored by pseudotime. (e) The UMAP plot shows the potential trajectories of all malignant epithelial cells colored by sample origin. (f) The UMAP plot shows the potential trajectories of all malignant epithelial cells colored by pseudotime state. (g) The UMAP plot shows the potential trajectories of all malignant epithelial cells colored by pseudotime.

To investigate the phenotypic characteristics of oxaliplatin-resistant clusters, we used GSVA to calculate the Hallmark gene set scores for each group of malignant epithelial cells (Figure 2a). The results revealed that, among all the malignant epithelial cells, Malignant_EP_04 exhibited the highest scores in the KRAS and EMT pathways, suggesting its potential for tumorigenesis and mesenchymal transformation. Additionally, we performed GSEA using GO and KEGG gene sets (Figure 2b,c) to study the functional roles of Malignant_EP_04. Notably, Malignant_EP_04 was significantly enriched in pathways related to mitochondrial energy supply and extracellular matrix regulation, both of which contribute to EMT transformation and oxaliplatin resistance [21–24].

To explore the transition from oxaliplatin-sensitive to oxaliplatin-resistant epithelial cells, we constructed a pseudotemporal developmental trajectory of tumor epithelial cells using single-cell data (Figure 2d). The tumor epithelial cells were classified into five developmental states (Figure 2f). Further analysis revealed that the tumor epithelial cells underwent two rounds of cell fate decisions, with some eventually differentiating into epithelial cells post-CT (Figure 2e). Oxaliplatin-resistant epithelial cells occupied the middle stage of the trajectory (Figure 2g), displaying characteristics of an overactive state. This strongly suggests that the Malignant_EP_04 subcluster may acquire oxaliplatin resistance through EMT.

### 3.3. Identification of CAF Subclusters

**Figure 3:**
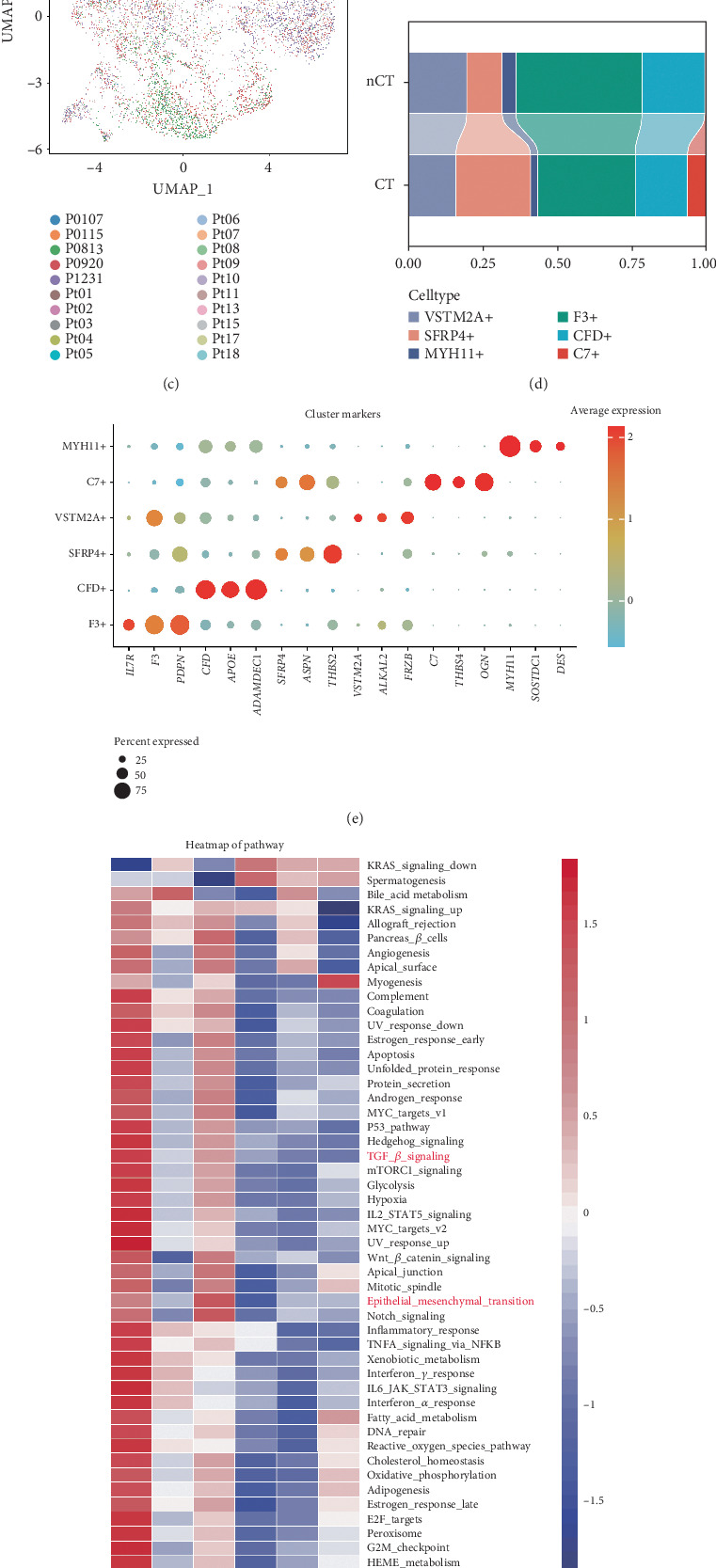
Phenotypic identification of tumor-associated fibroblasts. (a) UMAP plots of CAFs, colored by sample origin (CT and nCT). (b) UMAP plots of CAFs, colored according to CAF subtype. (c) UMAP plots of CAFs, colored by patients. (d) The bar chart shows the proportion difference of CAFs between the CT and nCT groups. (e) The dot plot shows the expression of marker genes for each subtype of CAFs. (f) The heatmap shows hallmark pathway enrichment in subtypes of CAFs. (g) The dot plot shows the KEGG enrichment of each subtype of CAFs.

Malignant_EP_04 exhibited significant extracellular matrix interactions. To characterize the changes in tumor-associated fibroblasts (CAFs) under different CT conditions, we performed subcluster clustering of fibroblasts (Figure 3a), dividing the CAFs into six subclusters (Figure 3b). These subclusters were named according to their primary marker genes: F3+, CFD+, SFRP4+, VSTM2A+, C7+, and MYH11+ (Figure 3e). Further analysis revealed that CT and nCT groups had different CAF compositions, with F3+ CAFs being predominant in both groups. VSTM2A+ CAFs and CFD+ CAFs were significantly reduced in the CT group, while SFRP4+ CAFs and C7+ CAFs were notably increased in the CT group (Figure 3d).

To further explore the pathway enrichment across CAF subclusters (Figure 3f), we calculated the Hallmark gene set scores for all CAF subclusters. The results showed that F3+ CAFs were significantly enriched in pathways related to inflammatory response, protein secretion, DNA repair, and hypoxia, which are closely associated with tumor stromal cell characteristics. SFRP4+ CAFs exhibited the highest scores in the KRAS and EMT pathways, suggesting their transcriptional similarity to the Malignant_EP_04 subcluster. Notably, both of these CAF subclusters displayed significant activation of the TGF-*β* pathway. Subsequently, we performed GSEA using the KEGG gene set (Figure 3g), and the results revealed that both subclusters were significantly enriched in pathways regulating the extracellular matrix. These findings demonstrate similar signaling pathway enrichment patterns between F3+ CAFs, SFRP4+ CAFs, and Malignant_EP_04, suggesting an EMT transformation between them.

### 3.4. Cell Communication Analysis Identifies TGF-*β* Pathway Involvement in EMT Process

**Figure 4:**
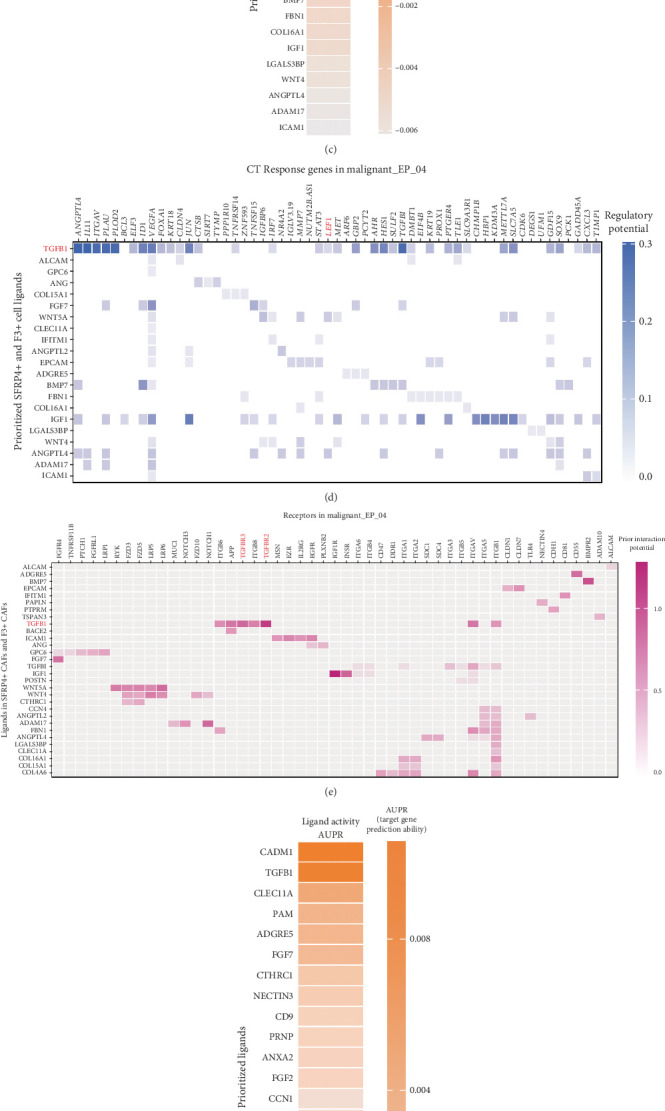
Intercellular communication of CAFs and malignant EPs. (a) The communication signal strength between CAFs and malignant EPs in the CT and nCT groups analyzed by CellChat. (b) Heatmap of the differences in communication signal intensity between CAFs and malignant EPs in the CT and nCT groups. (c) Heatmap of the Top 22 active ligands in SFRP4+ CAFs and F3+ CAFs sorted in accordance with AUPR values. (d) Heatmap of Top 22 active ligands and predicted target genes in Malignant_EP_04. (e) Heatmap of Top 22 active ligands and predicted receptors in Malignant_EP_04. (f) Heatmap of the Top 22 active ligands in F3+ CAFs sorted in accordance with AUPR values. (g) Heatmap of Top 22 active ligands and predicted target genes in SFRP4+ CAFs. (h) Heatmap of Top 22 active ligands and predicted receptors in SFRP4+ CAFs.

To further explore the core regulatory signaling pathways involved in the EMT process of F3+ CAFs, SFRP4+ CAFs, and Malignant_EP_04, we used the CellChat tool to evaluate the cell communication between malignant epithelial cell subclusters and CAF subclusters. The results showed that, compared to the nCT group, the Malignant_EP_04 subcluster in the CT group exhibited significantly stronger signal input, with F3+ CAFs and SFRP4+ CAFs being the major signal donors and receivers among CAFs (Figure 4a). The cell communication heatmap revealed notable differences between CT and nCT groups, with F3+ CAFs showing the most significant changes in communication quantity and MYH11+ CAFs displaying the largest differences in communication intensity (Figure 4b).

Additionally, we used the NicheNet tool to predict the potential ligand-receptor interactions and target gene relationships between F3+ CAFs, SFRP4+ CAFs, and Malignant_EP_04. The heatmap of the Top 30 most active ligands secreted by F3+ CAFs and SFRP4+ CAFs showed that TGFB1 exhibited extremely high activity in both groups (Figure 4c), with TGFB1 being a key ligand in the TGF*β* pathway. Further analysis indicated that TGFB1, secreted by F3+ CAFs and SFRP4+ CAFs, had the highest regulatory potential on the Malignant_EP_04 subcluster (Figure 4d). Furthermore, analysis of the potential receptors for the Top 30 ligands in Malignant_EP_04 revealed that TGFBR2 and TGFBR3, which are receptors for TGFB1, had a high probability of interaction in Malignant_EP_04 (Figure 4e).

These findings suggest that TGFB1, secreted by F3+ CAFs and SFRP4+ CAFs, binds to TGFBR2 and TGFBR3 receptors on Malignant_EP_04, thereby activating the TGF-*β* pathway.

Given that the expression of TGFB1 is higher in F3+ CAFs compared to SFRP4+ CAFs (Figure 3c), we conducted a similar analysis to identify potential ligands, receptors, and target genes between F3+ CAFs and SFRP4+ CAFs. The results showed that TGFB1 had the highest ligand activity in SFRP4+ CAFs (Figures 4f,g), binding to TGFBR1, TGFBR2, and TGFBR3 receptors (Figure 4h), regulating SFRP4+ CAFs. This suggests that F3+ CAFs can also regulate SFRP4+ CAFs by secreting TGFB1.

In conclusion, F3+ CAFs, by secreting TGFB1, interact with TGFBR2/TGFBR3 receptors on both SFRP4+ CAFs and Malignant_EP_04, enhancing TGF-*β* pathway signaling and ultimately driving EMT in Malignant_EP_04 towards SFRP4+ CAFs, which leads to oxaliplatin resistance.

### 3.5. LEF1 as a Specific Transcription Factor for Oxaliplatin Resistance Subcluster

**Figure 5:**
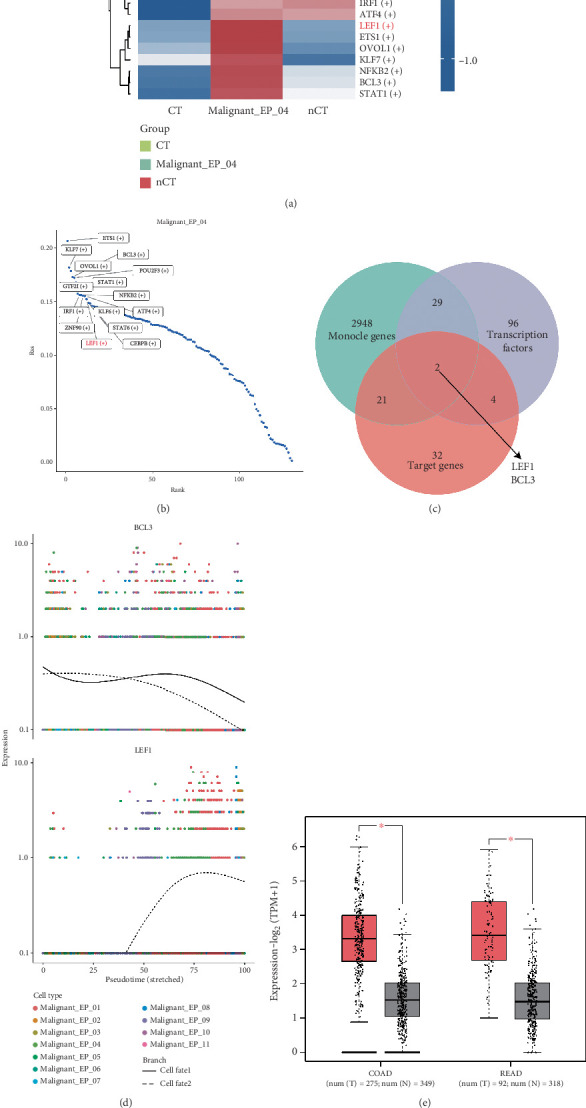
Scenic analysis revealed that LEF1 is the key transcription factor driving EMT in Malignant_EP_04. (a) The heatmap shows the differences in transfer factors among CT, nCT, and Malignant_EP_04. (b) Cluster-specific transcription factors for Malignant_EP_04. (c) Venn diagram of the genes driving cell speed changes, specific transcription factors, and predicted target genes in Malignant_EP_04. (d) Scatter plot of BCL3 and LEF1 expressions varying with pseudotime, with different colors representing different cell types. (e) The expression differences of LEF1 in cancer and adjacent tissues of COAD and READ were plotted using GEPIA2. (f) The KM curve of OS for the high-expression group and low-expression group of LEF1 was plotted using Kaplan–Meier Plotter. (g) The KM curve of DFS for the high-expression group and low-expression group of LEF1 was plotted using Kaplan–Meier Plotter.

We used the pySCENIC tool to infer the transcription factors for all malignant epithelial cells and visualized a heat map based on their specificity (Figure 5a). The key transcription factors that could potentially determine the fate of Malignant_EP_04 cells were ranked according to their regulatory specificity scores (Figure 5b).

To identify the specific target genes downstream of the TGFBR2/TGFBR3 pathway in Malignant_EP_04, we cross-referenced the key genes from the monocle branch, the 96 specific transcription factors in Malignant_EP_04, and the top 32 most likely target genes influenced by TGFB1. Ultimately, we identified two specific transcription factors: LEF1 and BCL3 (Figure 5c). We then plotted gene expression at Pseudotime Branch Point 2 in malignant epithelial cells, which revealed that BCL3 exhibited a decreasing expression trend, while LEF1 showed an increasing expression trend (Figure 5d). Compared to BCL3, LEF1 displayed more significant regulatory potential in relation to the TGFB1 ligand in the Malignant_EP_04 subcluster.

To elucidate the biological role of LEF1 in CRC, we observed that LEF1 expression was significantly upregulated in CRC compared to normal colon or rectal tissues, suggesting that LEF1 may promote CRC development [25] (Figure 5e). Moreover, analysis of multiple datasets from the Kaplan–Meier (KM) survival analysis indicated that patients with high LEF1 expression had worse overall survival and disease-free survival rates [26, 27] (Figures 5f,g).

### 3.6. Knockdown of LEF1 Inhibits EMT in Oxaliplatin-Resistant CRC Cells

**Figure 6:**
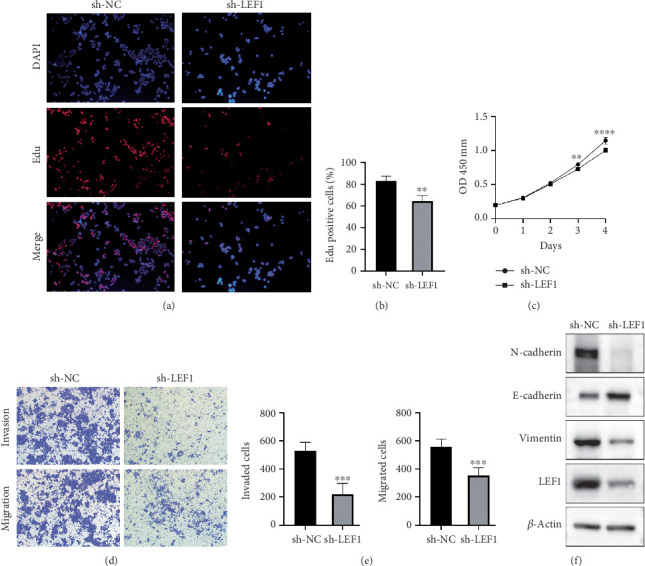
In vitro functional verification of the transcription factor LEF1. (a) Edu assay of sh-LEF1 in oxaliplatin-resistant HCT-116 cells. (b) The Edu assay compared the proliferation of the sh-LEF1 group and the control group in oxaliplatin-resistant HCT-116 cells. (c) The proliferation of LEF1 knockdown cells was assessed using the CCK8 assay. (d, e) The migration and invasion abilities of LEF1 knockdown cells were detected by Transwell assay. (f) The WB results revealed the expression of key EMT proteins in cells after LEF1 knockdown.

To further explore the biological function of LEF1 in CRC, we conducted a series of cell function experiments. First, we successfully established a LEF1 knockdown cell model in oxaliplatin-resistant HCT-116 cells using shRNA plasmids. Edu staining results showed that the number of Edu-positive cells was significantly reduced in the sh-LEF1 group (Figure 6a). Compared to the sh-NC group, the knockdown of LEF1 notably inhibited the proliferative activity of HCT-116 cells (Figure 6b). The CCK-8 assay further confirmed that LEF1 knockdown significantly reduced the proliferation rate of HCT-116 cells (Figure 6c). Transwell assays demonstrated that the knockdown of LEF1 effectively suppressed the migration and invasion ability of HCT-116 cells (Figures 6d,e).

Previous bioinformatics analysis indicated that LEF1 may promote EMT progression in drug-resistant tumor cells. To confirm this, we performed western blot analysis and found that, after LEF1 knockdown, the expression of E-cadherin was significantly upregulated, while the levels of N-cadherin and Vimentin were significantly reduced in oxaliplatin-resistant HCT-116 cells (Figure 6f). Therefore, the knockdown of LEF1 can inhibit the EMT process in oxaliplatin-resistant CRC cells.

## 4. Discussion

CRC is one of the leading causes of cancer-related mortality worldwide, and its therapeutic effectiveness is limited by CT resistance, particularly during oxaliplatin treatment [28]. CAFs are critical components of the TME, and they interact with tumor cells by secreting cytokines, matrix metalloproteinases, growth factors, and other molecules, thereby promoting tumor progression and resistance [29]. Previous studies have shown that CAFs activate EMT through signaling pathways such as TGF-*β*, Wnt, and Notch, enhancing the migratory, invasive, and chemoresistant properties of tumor cells [30–32]. In CRC, EMT is considered one of the key driving factors of cancer progression and metastasis, and it has been demonstrated to be closely related to CT resistance [33, 34]. The EMT process causes cancer cells to transition from an epithelial phenotype to one with mesenchymal characteristics, which are associated with increased self-renewal ability, migration, and drug resistance [35, 36]. Therefore, understanding the molecular mechanisms of oxaliplatin resistance, particularly the role of EMT in resistance, is of significant clinical importance.

In this study, we explored the mechanism by which EMT drives oxaliplatin resistance in CRC at the single-cell level. Using scRNA-seq, we identified a population of epithelial cells, termed Malignant_EP_04, which displayed characteristics of oxaliplatin resistance. This population exhibited prominent EMT features and further differentiated into SFRP4+ CAFs under the influence of TGFB1. Through pySCENIC and pseudotime analysis of key genes, we pinpointed LEF1 as a crucial transcription factor driving the EMT transition. Subsequently, we examined the receptor-ligand and target gene interactions between three subpopulations: F3+ CAFs, SFRP4+ CAFs, and Malignant_EP_04. Our findings suggest that F3+ CAFs may secrete TGFB1, which activates the TGFBR2/TGFBR3 receptors in SFRP4+ CAFs and Malignant_EP_04 cells. This leads to an increase in LEF1 activity within the tumor cells, driving Malignant_EP_04 cells to undergo EMT and transdifferentiate into SFRP4+ CAFs. In vitro validation experiments demonstrated that silencing LEF1 significantly reduced the proliferation and invasion of oxaliplatin-resistant CRC cell lines and inhibited their EMT progression. Recent research indicates that factors driving EMT could serve as promising prognostic biomarkers or therapeutic targets [37, 38]. Therefore, our findings may open new avenues for identifying drug targets aimed at overcoming CT resistance in CRC.

LEF1 is a key transcription factor in the Wnt/*β*-catenin signaling pathway and plays a critical role in various tumor types. In esophageal cancer, LEF1 activates the ERK/MAPK signaling pathway via the Id3/HRAS axis, thereby promoting the development and progression of esophageal squamous cell carcinoma (ESCC) [39]. Additionally, as a transcription factor, LEF1 enhances the oncogenic potential of ESCC and activates the TGF-*β* signaling pathway [40]. In gastric cancer, ASPN suppresses apoptosis in gastric cancer cells by regulating *β*-catenin-independent LEF1-mediated gene transcription [41]. Conversely, selective inhibition of LEF1 induces apoptosis through the Wnt/*β*-catenin pathway, thereby suppressing gastric cancer cell growth [42]. In liver cancer, LEF1 activates both the Wnt/*β*-catenin and NOTCH signaling pathways, enhancing the tumorigenic capacity of hepatocellular carcinoma (HCC) cells [43, 44]. Targeting the LEF1-mediated EMT process can reverse lenvatinib resistance in liver cancer cells [45]. In CRC, increased LEF1 expression is associated with poorer patient prognosis. [40, 46–48]. LEF1 generally functions as an upstream transcription factor, influencing key cellular processes such as proliferation, migration, invasion, and remodeling of the TME by regulating the transcription of downstream target genes. Under normal physiological conditions, LEF1 regulates the Wnt pathway by binding to *β*-catenin. However, when this pathway is dysregulated, elevated expression of LEF1 can drive cancer initiation and progression [49]. In CRC, LEF1's role is particularly linked to the TGF-*β* signaling pathway [50, 51]. Typically, TGF-*β* signaling prevents *β*-catenin from binding to TCF/LEF, which in turn suppresses the transcription of oncogenes such as c-myc, thereby inhibiting cell proliferation and migration [48]. In contrast, when LEF1 is overexpressed, it replaces TCF-4 to bind with *β*-catenin, leading to enhanced c-myc expression [50]. As a result, even when TGF-*β* signaling is activated, *β*-catenin is not removed, and c-myc transcription is not inhibited. Consequently, high LEF1 expression enables CRC cells to resist the inhibitory effects of TGF-*β* signaling, further promoting EMT and enhancing tumor invasiveness and metastasis. In CRC, LEF1 orchestrates glycolytic reprograming and EMT-associated phenotypic transformation, forming a synergistic network with mitochondrial dysfunction that collectively drives CT resistance in CRC. Specifically, the LEF/TCF-*β*-catenin complex sustains the glycolytic phenotype of CRC cells by regulating the transcription of the SLC16A1 gene and upregulating monocarboxylate transporter 1 (MCT-1) [52]. Moreover, ASCL2 enhances CT resistance in CRC cells by upregulating LEF1 transcription [53]. The sensitivity of CRC cells to oxaliplatin, 5-fluorouracil (5-FU), and irinotecan can be enhanced by targeted inhibition of LEF1 [54].

Although this study provides valuable insights into the resistance mechanisms, several challenges remain. For instance, effectively targeting the critical factor LEF1 in the EMT process and developing personalized targeted therapies for clinical application remain central to future research. Currently, some drugs are being investigated for their potential to inhibit LEF1-mediated stem cell characteristics in CRC. For example, niclosamide interferes with the binding of LEF1 to the DCLK1-B promoter, thereby blocking the transcription of DCLK1-B. The depletion of DCLK1-B weakens cancer stemness, reduces cell survival capacity, and increases apoptosis levels, ultimately enhancing the sensitivity of CRC cells to radiotherapy and CT. However, their efficacy still requires further validation through clinical trials and animal models [55].

Overall, our study highlights that in CRC, the TGF-*β* signaling pathway can elevate LEF1 transcriptional activity, promoting the EMT process in cancer cells. This process not only increases the migration and invasiveness of these cells but also enhances their resistance to CT, particularly oxaliplatin. Moreover, LEF1 may contribute to CRC metastasis and drug resistance by enhancing cancer cells' stem cell-like properties. Our findings provide new insights into the molecular mechanisms underlying CT resistance in CRC and offer significant experimental support for the development of LEF1 as a potential drug target. Future research should further explore the interactions between LEF1 and other TME factors to understand its universal role in various cancers, thereby driving the development of next-generation cancer therapies.

## 5. Conclusion

In conclusion, our study utilizing scRNA-seq reveals critical insights into the molecular mechanisms of oxaliplatin resistance in CRC, specifically through the activation of EMT in both epithelial and CAF subpopulations. By identifying specific cellular clusters and signaling pathways involved in resistance, we provide a clearer understanding of the TME's role in CT failure. These findings emphasize the potential of functional immune profiling and scRNA-seq in the discovery of reliable predictive and prognostic biomarkers, which are essential for refining treatment strategies in CRC. Further investigation into immune modulation within the TME may lead to the development of novel immune-based therapies that can overcome current therapeutic challenges and improve patient outcomes in CRC. The integration of these molecular insights with immuno-oncology approaches holds great promise for advancing precision medicine in the treatment of CRC.

## Data Availability

The data that support the findings of this study are openly available in Gene Expression Omnibus at https://www.ncbi.nlm.nih.gov/geo/, reference numbers GSE225857 and GSE245552.
